# Association Between Genetic Variation in *FOXO3* and Reductions in Inflammation and Disease Activity in Inflammatory Polyarthritis

**DOI:** 10.1002/art.39760

**Published:** 2016-10-27

**Authors:** Sebastien Viatte, James C. Lee, Bo Fu, Marion Espéli, Mark Lunt, Jack N. E. De Wolf, Lily Wheeler, John A. Reynolds, Madhura Castelino, Deborah P. M. Symmons, Paul A. Lyons, Anne Barton, Kenneth G. C. Smith

**Affiliations:** ^1^ University of ManchesterManchesterUK; ^2^ University of Cambridge School of Clinical MedicineCambridgeUK; ^3^ University of Manchester, Manchester, UK, and University College LondonLondonUK; ^4^ UMR 996, Inflammation, Chemokines, and Immunopathology, INSERM, Université Paris‐Sud, Université Paris‐SaclayClamartFrance; ^5^ NIHR Manchester Musculoskeletal Biomedical Research Unit, Central Manchester NHS Foundation Trust, Manchester Academic Health Science CentreManchesterUK

## Abstract

**Objective:**

Genetic variation in *FOXO3* (tagged by rs12212067) has been associated with a milder course of rheumatoid arthritis (RA) and shown to limit monocyte‐driven inflammation through a transforming growth factor β1–dependent pathway. This genetic association, however, has not been consistently observed in other RA cohorts. We sought to clarify the contribution of *FOXO3* to prognosis in RA by combining detailed analysis of nonradiographic disease severity measures with an in vivo model of arthritis.

**Methods:**

Collagen‐induced arthritis, the most commonly used mouse model of RA, was used to assess how *Foxo3* contributes to arthritis severity. Using clinical, serologic, and biochemical methods, the arthritis that developed in mice carrying a loss‐of‐function mutation in *Foxo3* was compared with that which occurred in littermate controls. The association of rs12212067 with nonradiographic measures of RA severity, including the C‐reactive protein level, the swollen joint count, the tender joint count, the Disease Activity Score in 28 joints, and the Health Assessment Questionnaire score, were modeled longitudinally in a large prospective cohort of patients with early RA.

**Results:**

Loss of *Foxo3* function resulted in more severe arthritis in vivo (both clinically and histologically) and was associated with higher titers of anticollagen antibodies and interleukin‐6 in the blood. Similarly, rs12212067 (a single‐nucleotide polymorphism that increases *FOXO3* transcription) was associated with reduced inflammation, both biochemically and clinically, and with lower RA activity scores.

**Conclusion:**

Consistent with its known role in restraining inflammatory responses, *FOXO3* limits the severity of in vivo arthritis and, through genetic variation that increases its transcription, is associated with reduced inflammation and disease activity in RA patients, effects that result in less radiographic damage.

Like most autoimmune and inflammatory conditions, the course of rheumatoid arthritis (RA) is unpredictable and is highly variable between patients. While current guidelines advocate early and aggressive treatment for all patients in order to avoid irreversible joint damage and disability, this strategy will undoubtedly result in the overtreatment of patients with mild disease or those in whom the disease would have spontaneously entered remission. Accordingly, such patients are currently exposed to the risks and side effects of unnecessary immunosuppression. To overcome this limitation, a personalized management strategy would be necessary, which in turn, would rely on having a reliable method of predicting disease outcome. Currently, however, the predictive value of clinical or serologic markers is insufficient to guide treatment decisions and, accordingly, there is a clear and well‐recognized need for predictive biomarkers.

Genetics has been shown to play an important role in the development of RA through a series of large genome‐wide association studies (GWAS) 1, 2, 3, but its potential role in determining disease course following diagnosis has been less rigorously studied, even though radiographic outcome has been proposed to be partially genetically determined 4. Indeed, while many studies have reported genetic associations with outcome, virtually none of these outside the HLA reach genome‐wide significance, nor have the associations always been replicated 5, 6, 7, 8, 9.

We previously demonstrated that a single‐nucleotide polymorphism (SNP) in *FOXO3A* (rs12212067; T>G) was associated with prognosis in several tumor necrosis factor (TNF)–driven diseases and that this variant led to altered production of pro‐ and antiinflammatory cytokines by monocytes through a transforming growth factor β1 (TGFβ1)–dependent pathway 10. In RA specifically, we showed that minor allele carriage at rs12212067, which reduced the production of TNF, interleukin‐6 (IL‐6), IL‐1β, and IL‐8 and increased the production of IL‐10, was associated with a milder disease course (i.e., less joint damage over time) in large and well‐phenotyped cohorts of patients with early disease. Since that report, which also demonstrated that rs12212067 was associated with outcome in Crohn's disease and malaria, several smaller studies have attempted to replicate these associations. In Crohn's disease 11 and malaria 12, the reported associations were independently replicated, although in RA, a meta‐analysis of 5 smaller cohorts from the US and Europe failed to detect the same association signal 13. This situation is not unusual and in fact is similar to what occurred in the early era of GWAS when some of the first associations—many of which have since been replicated several times—were initially questioned by negative findings of follow‐up studies in small and consequently underpowered cohorts 14, 15, 16.

Here, we sought to better understand what contribution *FOXO3* might make to the clinical outcome of RA. To do this, we first investigated whether a role of *FOXO3* in altering arthritis severity was biologically plausible by examining the contribution of *FOXO3* to the severity of immune‐mediated arthritis in vivo. We then chose to examine a broader range of arthritis severity markers in a large cohort of patients with early RA and inflammatory polyarthritis to determine whether other associations might be present that would support (or indeed refute) the association of rs12212067 with outcome in RA.

## MATERIALS AND METHODS

### Collagen‐induced arthritis

To study the role of *FOXO3* in immune‐mediated arthritis in vivo, we used the collagen‐induced arthritis mouse model, the most commonly used animal model of RA, in which arthritis is induced by immunization with emulsified type II collagen in Freund's complete adjuvant (50 μl of a 4 mg/ml preparation administered intradermally). We compared the severity of the resulting arthritis in C57BL/6 mice harboring a missense mutation in the highly conserved forkhead DNA‐binding domain that abrogates the function of *Foxo3* (Foxo3a^MommeR1/MommeR1^ mice; hereinafter referred to as Foxo3a^–/–^ mice) 17, with the arthritis that developed in heterozygous (Foxo3a^+/–^) and wild‐type (Foxo3a^+/+^) littermates (all 8 weeks old). Arthritis severity was assessed using a standard scoring system 18. Serum anticollagen antibodies (Chondrex) and IL‐6 and TNF levels (R&D Systems) were assessed by enzyme‐linked immunosorbent assay on day 14. All experiments were performed according to the regulations of the UK Home Office Scientific Procedures Act (1986).

### Patient cohorts and genotyping

To further investigate the relationship between rs12212067 (a noncoding SNP within *FOXO3*) and disease severity in RA, we used the Norfolk Arthritis Register (NOAR), a primary care–based inception cohort of 4,293 patients with early inflammatory polyarthritis who were monitored prospectively from disease onset up to 20 years 19 (Supplementary Table 1, available on the *Arthritis & Rheumatology* web site at http://onlinelibrary.wiley.com/doi/10.1002/art.39760/abstract). Inflammatory polyarthritis patients who satisfied the American College of Rheumatology 1987 criteria for RA 20, which were applied cumulatively over the first 5 years of follow‐up, were designated as RA patients (n = 2,537) (see Supplementary Table 1).

Where data were available, we analyzed the association of genotype at rs12212067 with a range of RA severity indices, including radiographic measures (Larsen score [21], modified Larsen score, and presence of erosions, defined as a cortical break ≥2 mm), biologic measures (C‐reactive protein [CRP] level), patient‐reported outcomes (Health Assessment Questionnaire [HAQ] score), and disease activity scores (swollen joint count, tender joint count, and Disease Activity Score in 28 joints [DAS28] calculated using the swollen joint count, tender joint count, and CRP level). Genotyping of rs12212067 was performed on a custom‐designed genotyping chip (Illumina ImmunoChip) or using Sequenom MassArray technology. Quality control procedures for the ImmunoChip data were as described in the original report 2. Ethical approval for the NOAR study was given by the Norwich Research Ethics Committee, and all patients gave informed consent.

### Statistical analysis

In order to maximize the power for the study of outcome in RA and inflammatory polyarthritis, we incorporated multiple records per patient over time and considered all measures of disease outcome (or activity) as longitudinal continuous variables. All analyses were adjusted for intraindividual correlation, and a dominant model of association was used (in part due to the low frequency of minor allele homozygotes). To provide a positive control, we used the same method to analyze the shared epitope (SE), a 5–amino acid sequence motif in the HLA–DRβ chain that is known to be associated with a variety of measures of severe RA. The presence or absence of the SE was determined experimentally using the reverse dot‐blot method, as described previously 22. We used generalized linear latent and mixed modeling for measures of radiographic outcome 23, zero‐inflated negative binomial regression for the CRP level 24, and quantile (median) regression for the HAQ score, swollen joint count, tender joint count, and DAS28. See the Supplementary Methods (available on the *Arthritis & Rheumatology* web site at http://onlinelibrary.wiley.com/doi/10.1002/art.39760/abstract) for further details.

Because the directions of effect of the SE and the *FOXO3A* SNP are already known, 1‐tailed *P* values are reported, as in the original study 10. Adjustment for multiple testing and treatment strategies was performed where indicated. Treatment was integrated in the model as a time‐dependent dummy variable for the presence of treatment with any disease‐modifying antirheumatic drug at every follow‐up visit. The proportion of the variance of a specific trait explained by the *FOXO3A* SNP was calculated as follows. The model was run with and without the SNP. For each model, the correlation coefficient between the observed and predicted values was calculated. The difference between the squares of the correlation coefficients was used to quantify the proportion of the variance explained by the SNP. These analyses were performed using Stata version 11/13 software (StataCorp).

In the collagen‐induced arthritis experiments, comparison of the arthritis score between the Foxo3^–/–^, Foxo3^+/–^, and Foxo3^+/+^ mouse groups was performed using a two‐way analysis of variance, and comparison of biochemical or serologic parameters between genotypes was performed using Wilcoxon's matched pairs signed rank test. One‐tailed *P* values are presented in view of the specific hypothesis being tested. These analyses were performed using GraphPad Prism v6.04 software.

## RESULTS

**Figure 1:**
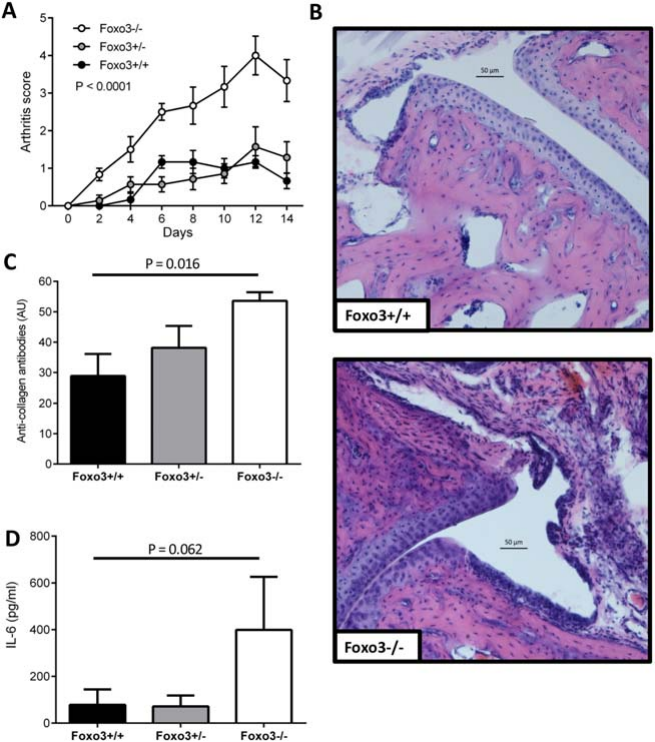
Loss of *Foxo3* activity and predisposition to more severe arthritis in Foxo3^–/–^, Foxo3^+/–^, and Foxo3^+/+^ littermate mice with collagen‐induced arthritis. **A,** Arthritis scores in the 3 mouse groups. Each limb was scored on a scale of 0–4 according to the method of Brand et al 18, and the scores were summed. **B,** Light microscopy of hematoxylin and eosin–stained sections of joints obtained on day 14 from Foxo3^+/+^ and Foxo3^–/–^mice, demonstrating joint space narrowing and synovial expansion. **C,** Serum anticollagen antibody titers on day 14. **D,** Serum levels of interleukin‐6 (IL‐6) on day 14. Results are representative of 2 experiments using a minimum of 6 mice per group. Values are the mean ± SEM. *P* values were determined by two‐way analysis of variance (**A**) or Wilcoxon's signed rank test (**C** and **D**).

To investigate how altered *FOXO3* function might influence the course of autoimmune arthritis in vivo, we first assessed the severity of collagen‐induced arthritis in mice carrying a missense mutation in the highly conserved forkhead DNA‐binding domain of *Foxo3*, which is known to abrogate its function. We found that carriage of the *Foxo3*‐null allele was associated with more‐severe arthritis. This was apparent from both the significantly higher arthritis scores (Figure 1A) and the joint histology findings, which demonstrated increased synovial expansion and cellularity and tissue edema (Figure 1B). In addition to assessing the clinical severity of arthritis, we also sought to compare the titers of anticollagen antibodies that were formed, as this enables a more direct evaluation of the immune response against type II collagen. These antibodies are also known to target epitopes within type II collagen that are shared by RA antibodies 25. We demonstrated that higher levels of anticollagen antibodies were present in mice with impaired or abrogated *Foxo3* function (Figure 1C). On day 14, we also assessed the production of IL‐6 and TNF, proinflammatory cytokines that are known to be important in the pathogenesis of collagen‐induced arthritis, and observed a nonsignificant trend toward higher IL‐6 levels in Foxo3^–/–^ mice as compared to either the wild‐type or heterozygous littermates (*P* = 0.06) (Figure 1D). Serum TNF was not detectable at this time point (data not shown).

In light of these results and our previous work that demonstrated an association between genotype at rs12212067 and the radiographic course of RA, we elected to examine other indices of disease severity in a large prospectively collected cohort of early RA patients. First, we considered other radiographic measures, as it has recently been suggested that the number of eroded joints may constitute a more accurate measure of joint damage 26. We confirmed that irrespective of the measure used, carriage of the minor allele at rs12212067 was associated with lower radiographic damage over time (i.e., lower Larsen score, lower modified Larsen score, and fewer erosions) (Table 1). In other words, patients with a milder disease course were more likely to carry the rs12212067 minor allele than those with a more severe disease course (the average minor allele frequency of rs12212067 in NOAR is 9.6%). The effect of *FOXO3A* rs12212067 on the Larsen score remained significant after adjustment for treatment (effect size equal to a reduction of 1.50 Larsen units for carriage of the G allele; *P* = 0.009) and was independent of the SE in a bivariate analysis (*P* = 0.002) (Table 1). However, although this association was significant, the proportion of the variance of the Larsen score explained by the carriage of rs12212067 was low (0.07%).

**Table 1:** Association of *FOXO3A* and the shared epitope with radiologic measures of disease outcome in patients with rheumatoid arthritis^a^

	Shared epitope		*FOXO3A* (rs12212067)	
	Effect size (95% CI)	*P*	Effect size (95% CI)	*P*
Larsen score				
Unadjusted	1.42 (0.50, 2.34)	0.0013	−1.50 (−2.73, −0.26)	0.0089
Adjusted for the shared epitope	–	–	−1.88 (−3.15, −0.60)	0.0020
Adjusted for treatment	–	–	−1.50 (−2.75, −0.25)	0.0094
Modified Larsen score	1.51 (0.66, 2.35)	0.00024	−1.65 (−2.80, −0.50)	0.0025
No. of eroded joints	0.36 (0.17, 0.54)	0.00012	−0.29 (−0.53, −0.06)	0.0073

a Effect size represents the change in Larsen score units or in the number of eroded joints. The modified Larsen score was obtained by recalculating the Larsen score using the presence of joint space narrowing (in the absence of any other modification) as 0 instead of 1, in order to reduce potential misclassification due to osteoarthritis. 95% CI = 95% confidence interval.

Given these data and the TGFβ1‐driven inflammatory pathway that *FOXO3* has been shown to regulate 10, we postulated that the effect of *FOXO3A* on radiographic outcome might be mediated by a reduction of inflammation and not by a direct effect on the bone, as has been suggested for other genetic markers of RA severity 5. We therefore examined the entire NOAR cohort to determine whether there was an association between rs12212067 genotype and nonradiographic measures of inflammation, disease activity, or outcome (including the CRP level, swollen joint count, tender joint count, the HAQ score, and DAS28). Data availability and the frequency and duration of follow‐up for these different measures varied and are shown in Supplementary Table 2 (available on the *Arthritis & Rheumatology* web site at http://onlinelibrary.wiley.com/doi/10.1002/art.39760/abstract).

We observed significant associations of rs12212067 genotype with the DAS28, swollen joint count, and CRP level and a nominally significant association with the HAQ score (*P* = 0.013, although the stringent bootstrap correction for interindividual correlation meant that the corrected *P* value was 0.11). Notably, there was also a consistent direction of effect across all of the variables, with minor allele carriage at rs12212067 generally associating with indices of milder disease, and with effect sizes that were of a comparable magnitude to those that were associated with the SE (Tables 1 and 2). However, the proportion of the phenotype variance explained by the carriage of rs12212067 remained low for nonradiographic measures of disease outcome (the proportion of the variance of DAS28 explained by only the SNP was 0.09%).

**Table 2:** Association of *FOXO3A* and the shared epitope with nonradiologic measures of disease outcome or activity in patients with inflammatory arthritis^a^

	Shared epitope		*FOXO3A* (rs12212067)	
	Odds ratio (95% CI)	*P*	Odds ratio (95% CI)	*P*
HAQ score	1.15 (1.06–1.25)	0.00019	0.94 (0.84–0.96)	0.11
DAS28	1.19 (1.02–1.38)	0.013	0.78 (0.66–0.93)	0.0029
SJC	1.22 (1.05–1.42)	0.0052	0.86 (0.73–1.03)	0.048
TJC	1.08 (0.80–1.45)	0.3	0.84 (0.59–1.20)	0.17
CRP	1.38 (1.09–1.72)	0.0032	0.74 (0.56–0.97)	0.016

a For outcome measures modeled with quantile regression, the values are the odds ratio (raised to the power of the observed coefficient), normal‐based 95% confidence interval (95% CI), and *P* value by 1‐tailed bootstrap method. For C‐reactive protein (CRP) modeled with zero‐inflated negative binomial regression, the results from the categorical (inflate) part of the model are reported: odds ratio (raised to the power of the observed coefficient for being non‐zero), 95% CI, and robust *P* value. HAQ = Health Assessment Questionnaire; DAS 28 = Disease Activity Score in 28 joints; SJC = swollen joint count; TJC = tender joint count.

The anti–cyclic citrullinated peptide (anti‐CCP) antibody status is a strong and well‐established predictor of disease severity 27, and the effect of the SE on RA severity is mainly mediated by anti‐CCP 23, 28. In order to investigate the influence of anti‐CCP status on the association of *FOXO3A* rs12212067, we adjusted the effect of rs12212067 for anti‐CCP status and performed a stratification analysis by anti‐CCP status (Table 3). Although the effect of the SE on disease severity and activity disappeared completely after adjustment or stratification, the effect of rs12212067 on the Larsen score or DAS28 remained significant irrespective of anti‐CCP status (the effect size adjusted for anti‐CCP was equal to a reduction of 1.26 Larsen units for carriage of the G allele (95% confidence interval −2.49, −0.03; *P* = 0.023), and the odds ratio for the DAS28 was 0.78 (95% confidence interval 0.67, 0.90; *P* = 4.3 × 10^−4^).

**Table 3:** Adjustment for, and stratification by, anti‐CCP status^a^

	Anti‐CCP positive		Anti‐CCP negative		Adjusted for anti‐CCP status	
	Effect size (95% CI)	*P*	Effect size (95% CI)	*P*	Effect size (95% CI)	*P*
Shared epitope						
Larsen score	1.05 (−1.45, 3.54)	0.205	0.23 (−0.61, 1.07)	0.295	0.16 (−0.80, 1.11)	0.373
DAS28	1.13 (0.85, 1.50)	0.205	1.08 (0.90, 1.28)	0.206	1.10 (0.95, 1.27)	0.104
*FOXO3A* (rs12212067)						
Larsen score	−1.82 (−4.90, 1.26)	0.123	−0.98 (−1.96, 0.01)	0.026	−1.26 (−2.49, −0.03)	0.023
DAS28	0.78 (0.60, 1.02)	0.033	0.80 (0.66, 0.96)	0.010	0.78 (0.67, 0.90)	4.3 × 10^−4^

a The association studies presented in Table 1 for the Larsen score in rheumatoid arthritis patients and in Table 2 for the Disease Activity Score in 28 joints (DAS28) in inflammatory polyarthritis patients were again performed either by adjusting for the anti–cyclic citrullinated peptide (anti‐CCP) status; that is, the analysis was restricted to anti‐CCP–positive disease or anti‐CCP–negative disease. Since the effect of the shared epitope is almost completely mediated by anti‐CCP, it disappears completely. However, the adjustment has no major influence on the association of *FOXO3A* rs12212067. Stratification decreases the sample size and therefore the power, which is likely to explain the lack of significance for the Larsen score in the smaller anti‐CCP–positive group. Overall, these results indicate that the biologic pathways that mediate the effect of the shared epitope and *FOXO3A* are different. *P* values are 1‐tailed. See Tables 1 and 2 for explanations of dimensions and interpretation of effect sizes. 95% CI = 95% confidence interval.

## DISCUSSION

FOXO3 is a transcription factor that has been linked to the regulation of immune responses through the use of systems biology 29 and knockout mouse models 30, and it has been shown to be overexpressed in blood and synovial leukocytes in RA 31. We have previously reported that minor allele carriage at rs12212067, a noncoding SNP in *FOXO3*, is associated with a milder course of several TNF‐driven diseases, including RA, and we have described a *FOXO3*‐dependent pathway that is regulated by this genetic variant and which modulates inflammatory responses in monocytes via TGFβ1 induction 10. This genetic association, however, was not detected in a follow‐up study by van Steenbergen and colleagues 13, which included 5 smaller RA cohorts and a mixture of prospective and retrospective patients as well as early and established RA. A nonsignificant trend toward a protective effect of the minor allele at rs12212067, which is consistent with our results, was observed in their meta‐analysis, however, particularly in the cohorts of early RA patients for whom multiple sets of radiographs were available 13. Nonetheless, given these apparently contradictory reports 10, 13, we sought to better understand what role, if any, *FOXO3* plays in influencing disease course in RA.

We first sought to establish whether a contribution of *FOXO3* to the course of immune‐mediated arthritis was biologically plausible, given that genetic associations alone do not establish biologic relevance to disease. Indeed, even though we had previously identified an inflammatory pathway that is modulated by genetic variation at rs12212067, this does not definitively prove that any association with radiographic outcome in RA is due to effects on inflammation. We therefore examined the role of *Foxo3* in immune‐mediated arthritis in vivo in an animal model that has previously been extensively and successfully used to delineate pathogenic mechanisms in RA and to test new therapies 18. Based on the results of these experiments, we then used detailed phenotype data from the largest cohort of early RA patients worldwide to assess associations with other measures of inflammation and disease severity.

We demonstrated that altered *Foxo3* function does modulate the severity of autoimmune arthritis in vivo, and we identified genetic associations with several other indices of inflammation and disease severity in RA patients. Together, these results strengthen the previously reported association of *FOXO3* with outcome in RA. Moreover, these data suggest that rather than having a direct effect on bone, the contribution of *FOXO3* to outcome in RA is likely to be by modulating inflammation (e.g., the CRP level) and disease activity (e.g., swollen joint count, DAS28), which would then lead to differences in radiographic outcome (e.g., Larsen score, erosions).

However, the effect of *FOXO3* is independent of the SE and of the presence of anti‐CCP antibodies, which is consistent with 2 different mechanisms of action for these 2 different genetic markers. Importantly, the size of the effects on radiographic damage and the DAS28, while statistically significant, are unlikely to be clinically useful in isolation (e.g., for applications such as predicting disease outcome), as the proportion of the phenotype variance explained by the *FOXO3* SNP alone is <0.1% (0.07% for the Larsen score and 0.09% for the DAS28).

This does not, however, preclude the inclusion of this SNP in a model that could incorporate several predictive factors (e.g. genetic, demographic, clinical, and environmental). In the future, such a model could comprise a set of several hundreds of SNPs of small effect sizes. With current technological advances and the commercialization of high‐throughput (chip‐based) genotyping platforms for clinical applications, this approach will become a realistic diagnostic/prognostic option. Moreover, irrespective of the utility of this SNP for predicting disease course, by studying the functional effects of such associations, it may be possible to uncover previously unappreciated pathways that are amenable to pharmacologic intervention and so develop better treatments.

Such a discrepancy between the effect size of an associated SNP and the therapeutic potential of targeting the underlying biology has already been observed in cardiovascular medicine, where genetic variants in *HMGCR* are associated with very modest changes in serum levels of low‐density lipoprotein (∼5% 32), but the protein product of this gene, hydroxymethylglutaryl‐coenzyme A reductase, is the pharmacologic target of the most effective drug treatment for hypercholesterolemia (statins) 33. An important caveat, however, is that the associations of rs12212067 with the indices of RA severity described herein have not yet been replicated, even though these indices were selected because of the a priori hypothesis that minor allele carriage at rs12212067 would be associated with milder disease. Accordingly, these associations should be examined further in larger and appropriately powered replication cohorts.

Since the advent of GWAS, it has been commonplace for the reporting of new genetic associations to be followed by several smaller, often underpowered, studies that attempt to replicate the reported associations, with varying degrees of success. In the present study, we confirmed that *FOXO3* plays an important role in determining the course of immune‐mediated arthritis in vivo, and we detected associations between genotype at rs12212067 and a range of RA severity measures. It is therefore important to consider why this association was not detected in a previous follow‐up study 13. One possibility is that the genetics and biology of disease outcome are genuinely different between the populations studied. However, given that both study populations were derived from the same ethnic ancestry, this seems less likely, and other possibilities need to be considered.

An alternative explanation is that the relatively small size of the individual cohorts in the replication study 13 may have limited their power to detect any effect. This is likely to have been further hampered by the considerable intercohort heterogeneity, both in terms of clinical phenotype (i.e., early versus established RA) and in terms of the available radiographic data. For example, in 50% of the cases, only a single set of radiographs were available. This meant that progression could not be assessed in a uniform way across all cohorts, being directly measured in some and extrapolated based on assumed linear disease progression in others, an assumption that is unlikely to be valid for most patients 34. Standardized phenotype definitions are critical for multicenter studies in order to prevent the introduction of site‐based bias(es) and to ensure that the final meta‐analysis is interpretable 35. Accordingly, we would contend that rather than disproving a role of *FOXO3* in the outcome of RA, this negative study actually reemphasizes the need for consistent definitions in replication studies, as has been highlighted by others 35.

Collectively, therefore, our data provide further support for the observation that an SNP which has not been associated with susceptibility to RA (minor allele frequency of 0.103 in 3,879 UK RA cases and 0.104 in 8,428 UK controls, with an odds ratio of 0.994 [95% confidence interval 0.910, 1.085); *P* = 0.89 [2]) is associated with disease outcome. This highlights possible new directions for candidate gene or pathway‐based studies in complex disease genetics and suggests that by identifying other genetic variants that associate with outcome, it may ultimately be possible to develop prognostic tests that have sufficient performance to guide treatment decisions. As with studies of disease susceptibility, however, such work will probably require large sample sizes and meta‐analyses to confirm associations at genome‐wide significance levels. International consortia will undoubtedly be critical in achieving this goal.

## AUTHOR CONTRIBUTIONS

All authors were involved in drafting the article or revising it critically for important intellectual content, and all authors approved the final version to be published. Drs. Barton and Smith had full access to all of the data in the study and take responsibility for the integrity of the data and the accuracy of the data analysis.

### Study conception and design

Viatte, Lee, Fu, Symmons, Lyons, Barton, Smith.

### Acquisition of data

Viatte, Lee, Espéli, De Wolf, Wheeler, Reynolds, Castelino, Symmons, Lyons, Barton, Smith.

### Analysis and interpretation of data

Viatte, Lee, Fu, Espéli, Lunt, De Wolf, Wheeler, Symmons, Lyons, Barton, Smith.

## Supporting information

Supporting Information
