# Supplementary material for: Association Between Genetic Variation in FOXO3 and Reductions in Inflammation and Disease Activity in Inflammatory Polyarthritis
Source: Arthritis Rheumatol. 2016 Oct 27;68(11):2629–36. doi: 10.1002/art.39760 (PMC5091631; doi:10.1002/art.39760)
Supplement: Supplementary file 1 — Supporting Information [file ART-68-2629-s001.docx]

**Genetic variation in FOXO3 is associated with reductions in inflammation and disease activity in inflammatory polyarthritis**

Sebastien Viatte ^1 †^, James C. Lee ^3,4 †^, Bo Fu ^1,5^, Marion Espéli ^6^, Mark Lunt ^7^, Jack N.E. De Wolf ^1^, Lily Wheeler ^1^, John A. Reynolds ^7^, Madhura Castelino ^7^, Deborah P.M. Symmons ^2,7^, Paul A. Lyons ^3,4^, Anne Barton ^1,2 †^*, Kenneth G.C. Smith ^3,4 †^*.

^1^ Arthritis Research UK Centre for Genetics and Genomics, Centre for Musculoskeletal Research, Faculty of Biology, Medicine and Health, Manchester Academic Health Science Centre, The University of Manchester, Manchester, Oxford Road, Manchester, M13 9PT, UK.

^2^ NIHR Manchester Musculoskeletal Biomedical Research Unit, Central Manchester NHS Foundation Trust, Manchester Academic Health Sciences Centre, Grafton Street, Manchester, M13 9WL, UK.

^3^ Cambridge Institute for Medical Research, University of Cambridge, Cambridge Biomedical Campus, Cambridge CB2 0XY, UK.

^4^ Department of Medicine, University of Cambridge School of Clinical Medicine, Addenbrooke’s Hospital, Cambridge CB2 0QQ, UK.

5 Administrative Data Research Centre for England, Farr Institute of Health Informatics Research – London, University College London, 222 Euston Road, London NW1 2DA and Population Policy and Practice Programme, Institute of Child Health, Faculty of Population Health Sciences, University College London, 30 Guilford Street, London WC1N 1EH.

^6^ UMR996 - Inflammation, Chemokines and Immunopathology -, Inserm, Univ Paris-Sud, Université Paris-Saclay, 92140, Clamart, France.

^7^ Arthritis Research UK Centre for Epidemiology, Centre for Musculoskeletal Research, Manchester Academic Health Science Centre, The University of Manchester, Oxford Road, Manchester, M13 9PT, UK.

^†^ These authors contributed equally to this work.

* Corresponding authors (KGCS and AB):

*KGCS:* Email: kgcs2@cam.ac.uk

Telephone: +44 (0)1223 336848

Address: Department of Medicine, University of Cambridge School of Clinical Medicine, Addenbrooke’s Hospital, Cambridge CB2 0QQ, UK.

*AB:* Email: Anne.Barton@manchester.ac.uk

Telephone: +44 (0)161 275 1638

Address: Arthritis Research UK Centre for Genetics and Genomics, Centre for Musculoskeletal Research, Manchester Academic Health Science Centre, University of Manchester, Manchester, Oxford Road, Manchester, M13 9PT, UK

**SUPPLEMENTAL DATA**

**Supplementary methods: Statistical analysis**

The longitudinal outcome variables studied here differ importantly with regards to the following characteristics: their distribution (including excess of zero values), the frequency and duration of data collection (see Supplementary Table 2), data availability/density at every follow-up, the kinetic of their median (see Supplementary Figure 1), mean and variance. Based on every variable’s specific summary statistics, availability and distribution over time, the dataset was censored at different time points for different outcome variables (for example, year 5 for DAS28 but year 10 for HAQ in NOAR) and the appropriate model was used for every outcome variable. GLLAMM was performed with discrete random effects and three latent classes. This implies a finite mixture of three-component normal distributions for the Larsen score or the number of erosions to capture their extra zero scores and their distribution skewness. Effect sizes are given as an increase in Larsen unit or in the number of erosions. Adjustment for age, disease duration and the square of them was performed to allow for a quadratic relationship between radiographic outcome and time/age. GLLAMM is usually inappropriate if too few time points are available or shows convergence problems if more than two polynomial terms for disease duration and age need to be fitted (non-convergence problem for cubic relationships or above). Therefore, when fitting polynoms of higher order was needed to improve model accuracy, we used quantile (median) regression or ZINB.[23] The entire cohort (see Supplementary Tables 1 and 2) was used to determine the order of the polynoms of age and disease duration; polynomial terms of increasing order were added sequentially, as long as they were significantly associated with disease outcome at the 0.05 level (Supplementary Figure 1). The standard error of parameter estimates in quantile regression was obtained with a bootstrap method with 500 iterations to stringently correct for intra-individual correlation.


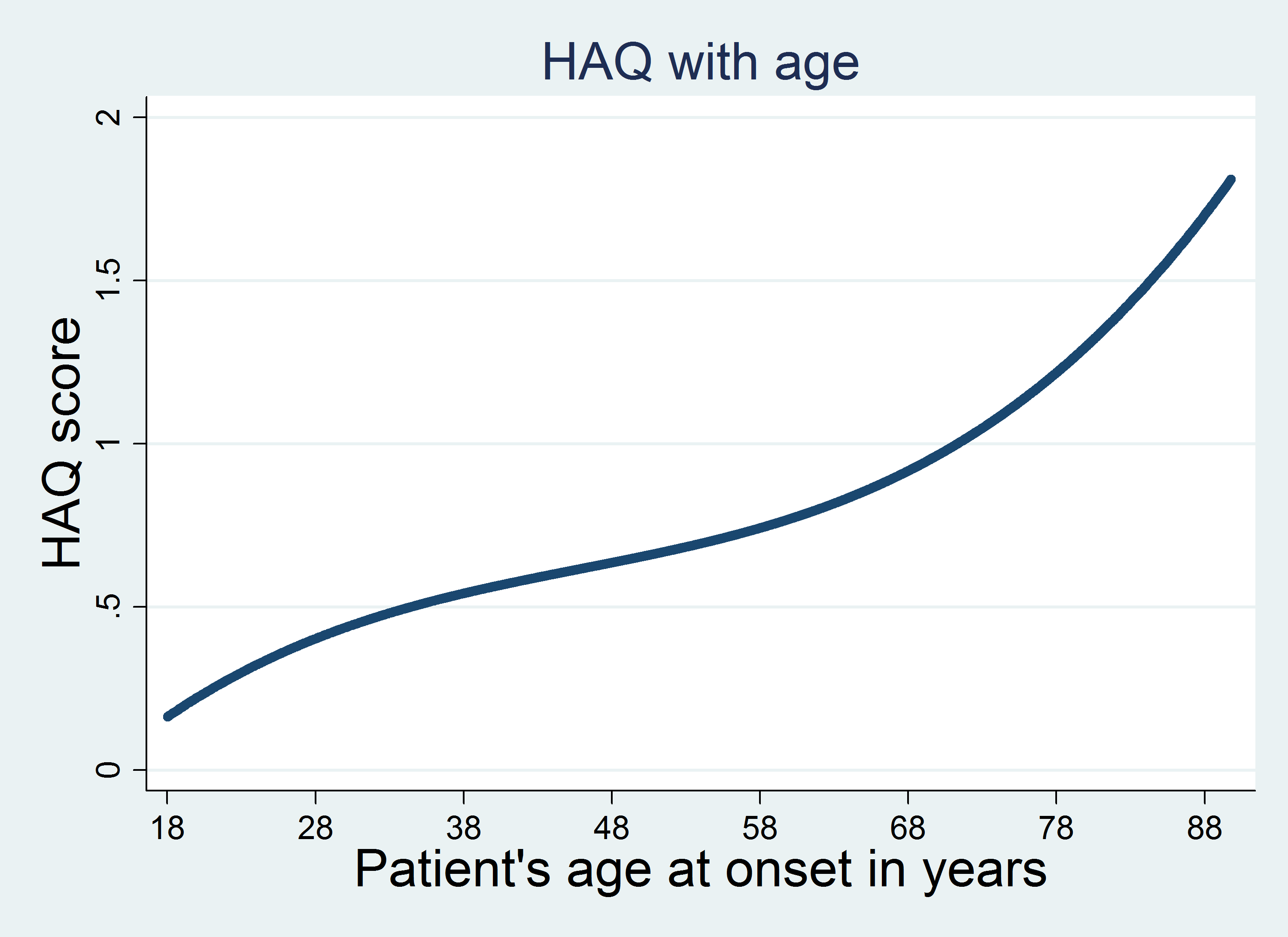


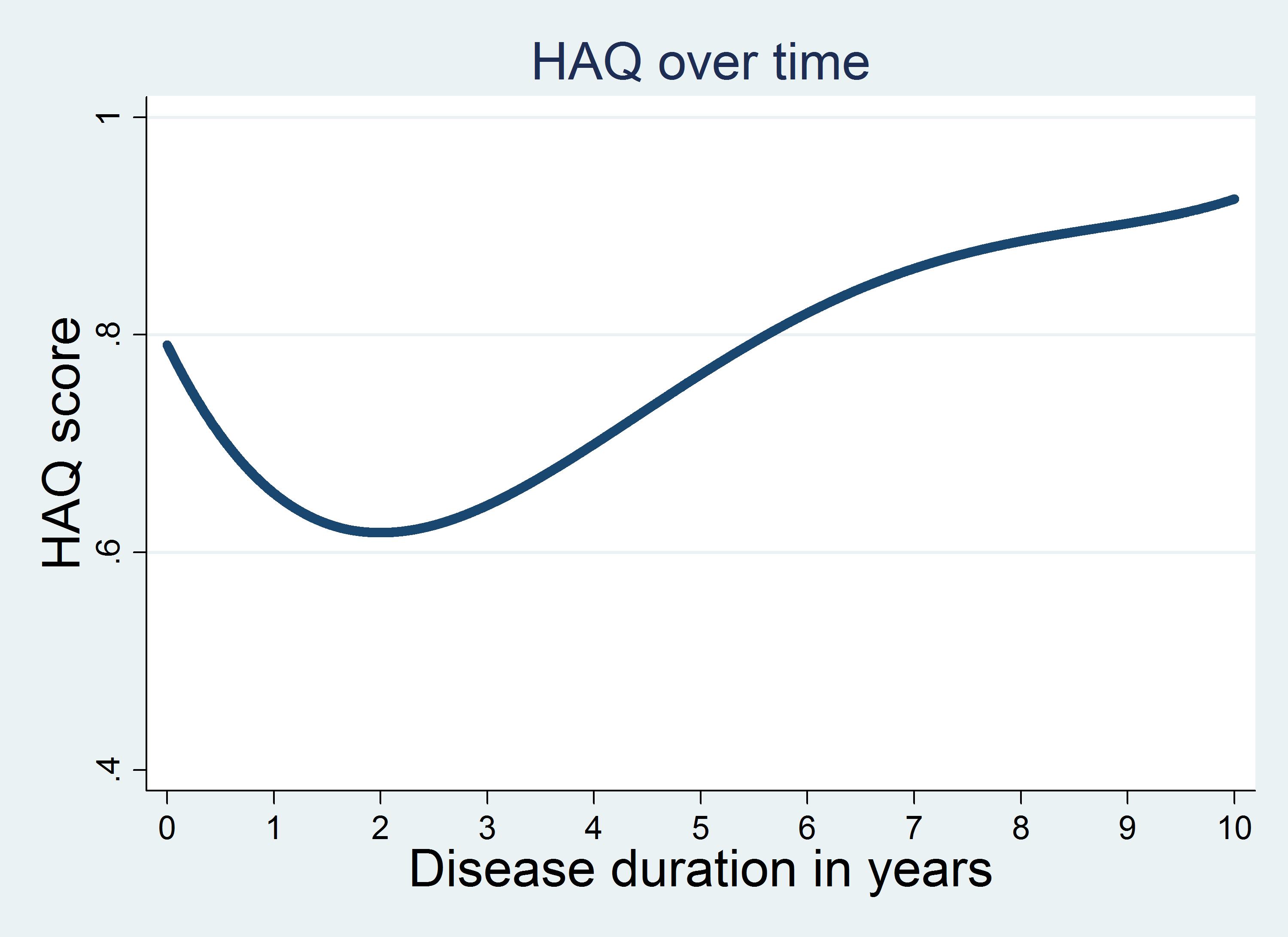


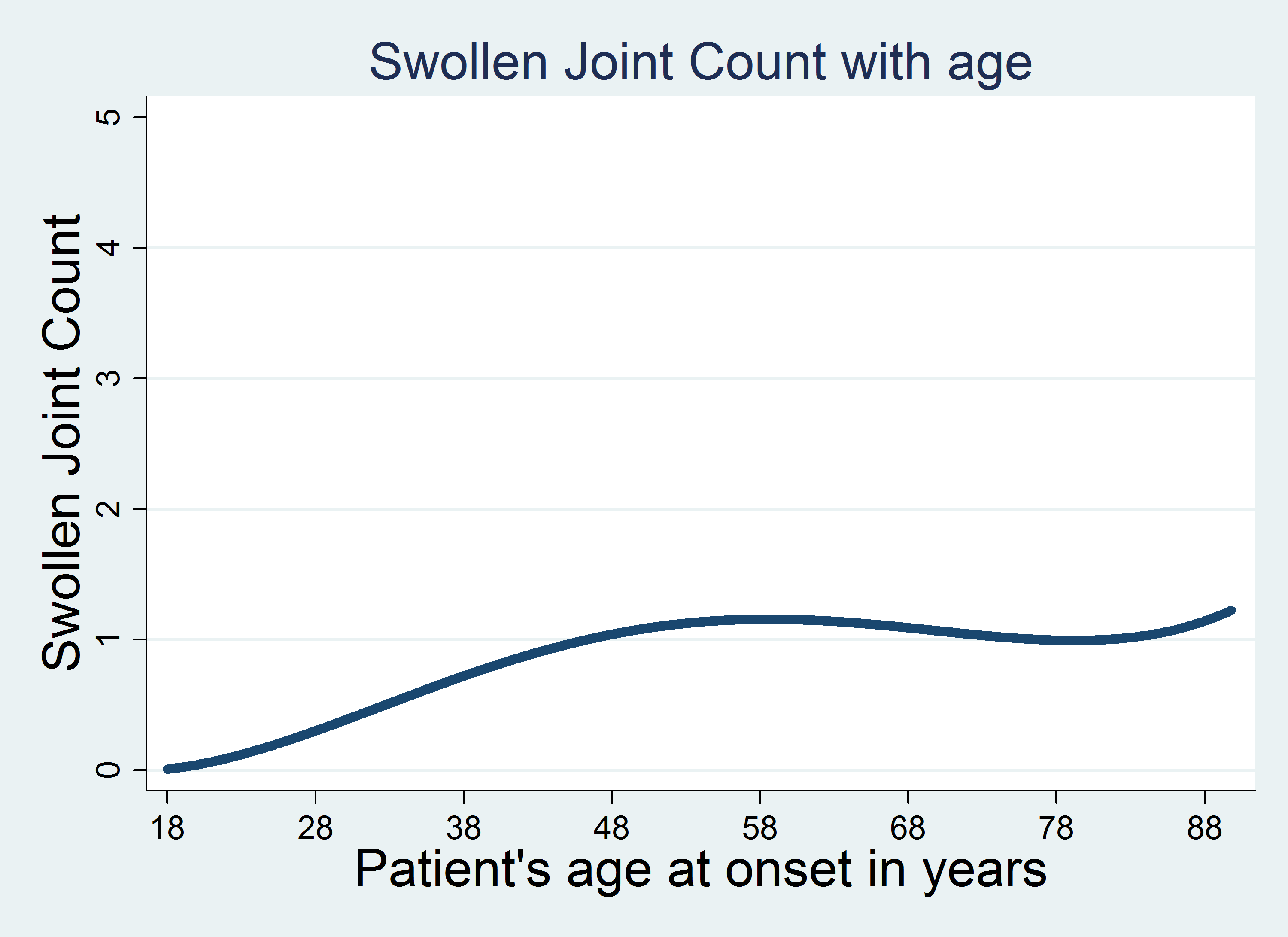


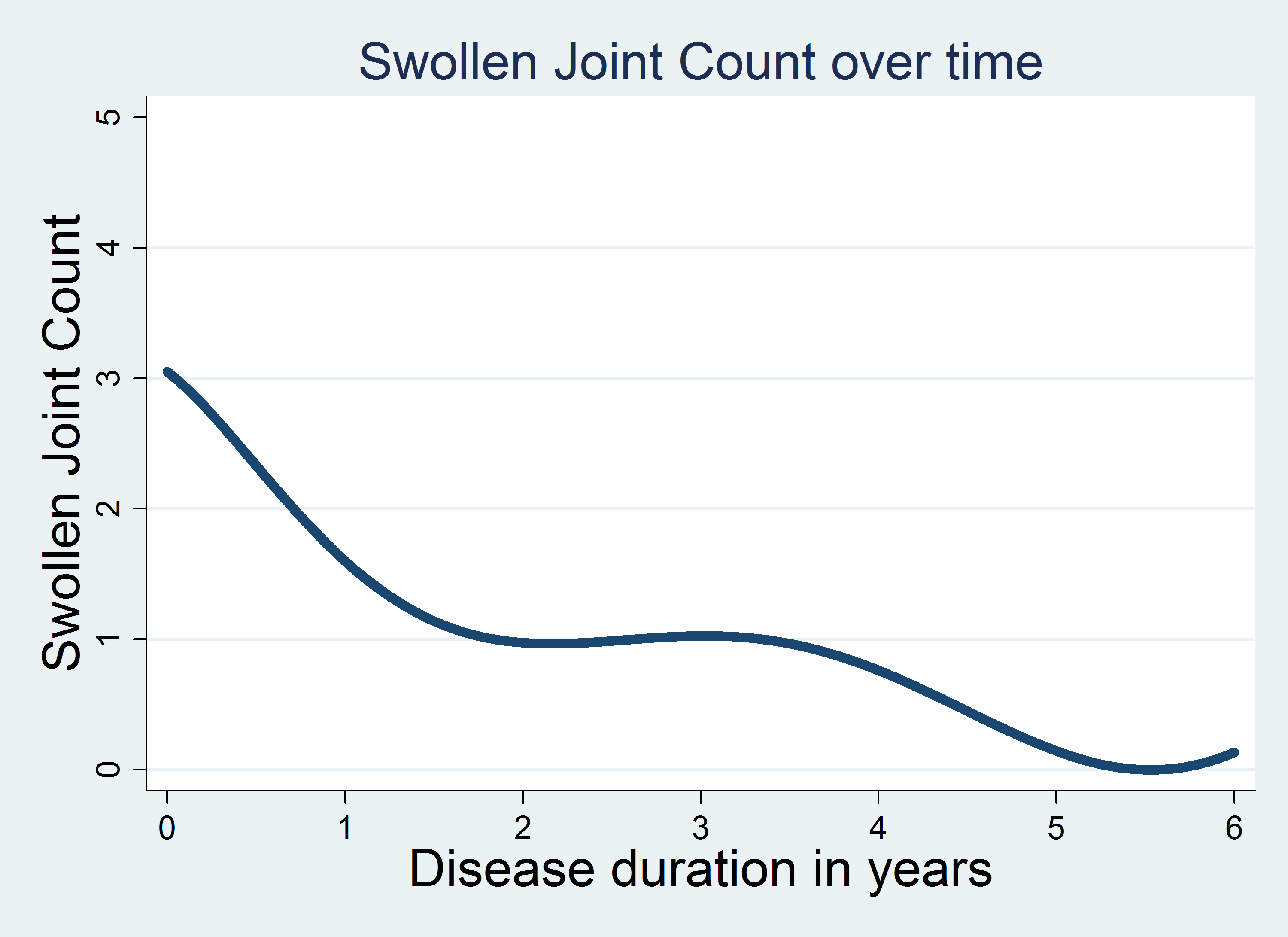


**Supplementary Figure 1. Modelling the effect of disease duration and age on measures of disease outcome**

Disease duration is the strongest predictor of disease severity and could therefore potentially mask the weak association of a genetic marker. The entire NOAR cohort (irrespective of the availability of genetic information) comprising 4293 patients followed up over time was used to determine the relationship between time or age and outcome variables. The predicted kinetic of the median HAQ score is shown here (upper panels), when a polynom of disease duration or age is used to model HAQ scores. Lower panels show the predicted Swollen Joint Count modelled with a polynom of disease duration or age. The same approach was used in ERAS.

|  |  | | |
| --- | --- | --- | --- |
|  | **Entire cohort** | **With genotype for the SE** | **With genotype for *FOXO3A*** |
| Number of patients with IP, baseline (year 5,10,15,20) | 4293 (2934, 892, 530, 175) | 2673 (2067, 785, 481, 157) | 2350 (1822, 710, 454, 158) |
| Total number of follow-ups | 24093 | 17132 | 15137 |
| Duration of follow-up in yrs, median (range) | 3 (0-20) | - | - |
| Number of follow-ups per patient, median (range) | 4 (1-13) | - | - |
| Number of patients with RA (%) | 2537 (59) | 1846 (69) | 1613 (69) |
| Number of female (%) | 2779 (65) | 1761 (66) | 1547 (66) |
| Age at symptom onset in yrs, median (IQR) | 55 (42-67) | 55 (43-67) | 55 (43-67) |
| Ever positive for anti-CCP (%) | 32 | 34 | 33 |
| On a DMARD at year 0, 1, 5, 10, 20 (%) | 31, 46, 48, 42, 37 | - | - |
| Larsen score at year 5, median (IQR) | 6 (0-22) | 6 (0-23) | 6 (0-23) |
| Patients with erosive disease at year 5(%) | 46 | 46 | 45 |
| HAQ score at year 5, median (IQR) | 0.8125 (0.125-1.625) | 0.875 (0.125-1.625) | 0.75 (0.125-1.625) |
| DAS28 at year 5, median (IQR) | 2.71 (2.02-3.75) | 2.73 (2.02-3.79) | 2.67 (2.02-3.64) |
| CRP at year 5, median (IQR) | 6.4 (0-14.2) | 6.4 (0-14.1) | 6.6 (0-14.7) |

**Supplementary Table 1. Characteristics of patients with inflammatory polyarthritis (IP)**

Erosive disease was defined as the presence of at least one erosive joint (Larsen score ≥ 2) according to Larsen: cortical break ≥ 2 mm. IP: inflammatory polyarthritis. RA: rheumatoid arthritis, i.e. patients who satisfied at any point the 1987 American College of Rheumatology (ACR) criteria applied cumulatively over the 5 first years of the follow-up. SE: shared epitope. DMARDs: disease modifying anti-rheumatic drugs. "-" not relevant. IQR: interquartile range. Anti-CCP: anticyclic citrullinated peptide.

|  | **Radiographic outcome** | **HAQ score** | **DAS28** | **SJC** | **TJC** | **CRP** |
| --- | --- | --- | --- | --- | --- | --- |
| Total number of patients | 1458 | 2347 | 2187 | 2350 | 2350 | 2200 |
| Total number of follow-ups (time points) | 2402 | 14294 | 3781 | 10806 | 10806 | 3971 |
| Duration of follow-up in yrs, mean (max) | 2.6 (10) | 4.1 (20) | 3.7 (15) | 3.3 (20) | 3.3 (20) | 3.8 (15) |
| Number of follow-ups per patient, mean (range) | 1.5 (1-5) | 4.4 (1-13) | 1.6 (1-4) | 3.1 (1-8) | 3.1 (1-8) | 1.6 (1-4) |

**Supplementary Table 2. Data availability for longitudinal modelling of different measures of disease outcome or activity**

Characteristics of NOAR patients are shown for the whole cohort (inflammatory polyarthritis - IP), irrespective of the availability of genotype information. The duration of follow-up and frequency of assessment, therefore the total number of time points, vary widely between different measures of disease outcome. For example, a max. of 4 time points per patients over 15 years is available to model DAS28, while some patients will have had their HAQ score measured 13 times over 20 years. The data presented in this table is used to model the effect of disease duration or age on different measures of disease outcome or activity (see Supplementary figure 1): radiographic outcome can be either the presence of erosive disease, the number of erosive joints or the Larsen score; SJC (swollen joint count); TJC (tender joint count).
